# Switching the hypoxic response on and off in plants

**DOI:** 10.1093/jxb/erag101

**Published:** 2026-02-24

**Authors:** Simone Castellana, Emma Olmi, Luca Brunello, Elena Loreti, Pierdomenico Perata

**Affiliations:** PlantLab, Institute of Plant Sciences, Sant’Anna School of Advanced Studies, via Guidiccioni 8/10, San Giuliano Terme, Pisa, Italy; PlantLab, Institute of Plant Sciences, Sant’Anna School of Advanced Studies, via Guidiccioni 8/10, San Giuliano Terme, Pisa, Italy; PlantLab, Institute of Plant Sciences, Sant’Anna School of Advanced Studies, via Guidiccioni 8/10, San Giuliano Terme, Pisa, Italy; Institute of Agricultural Biology and Biotechnology, National Research Council, via Moruzzi 1, Pisa, Italy; PlantLab, Institute of Plant Sciences, Sant’Anna School of Advanced Studies, via Guidiccioni 8/10, San Giuliano Terme, Pisa, Italy; Nagoya University, Japan

**Keywords:** Arabidopsis, hypoxia, low oxygen, oxygen sensing

## Abstract

Hypoxia significantly impacts plant metabolism and growth by disrupting mitochondrial respiration, and oxygen sensing plays a vital role in regulating responses to low oxygen conditions. Plants sense oxygen through the N-degron pathway, involving plant cysteine oxidases (PCOs) that oxidize the ethylene response factors belonging to group VII (ERF-VIIs), leading to their degradation under normoxia. Under hypoxic conditions, PCO activity decreases, stabilizing ERF-VII proteins and activating the transcription of hypoxia-responsive genes to adapt to oxygen limitation. Recent research highlights additional factors, including the MBR1/MED25 complex, ERF-VII phosphorylation, and the integration of energy and oxygen signals via the target of rapamicin pathway, which fine-tune the hypoxic response. Upon reoxygenation, PCOs restore activity and degrade ERF-VII, but this degradation is delayed, possibly due to reactive oxygen species inhibiting PCO function. Repressive factors such as HRA1 and ORA59 also modulate ERF-VII activity to suppress HRG expression. The response of the plant to hypoxia also involves a sophisticated network of molecular signals, including calcium signalling and the redox-modulatory role of phytoglobins and nitric oxide. Despite significant progress, much remains unknown about plant hypoxia, as its complex, spatiotemporal nature affects not only environmental adaptation but also development and plant–microbe interactions, necessitating intricate regulatory mechanisms.

## Teaser

Plants regulate their hypoxic response by activating signalling pathways to adjust gene expression and turning them off when oxygen levels rise, with fine-tuning through signalling and protein modifications.

## Introduction

Hypoxia is a condition that profoundly impacts plants. In the absence of oxygen, metabolism is significantly altered due to the cessation of mitochondrial respiration, and oxygen sensing plays a crucial role in regulating plant growth, development, and plant–microbe interactions (Loreti and Perata, 2020; van Veen *et al.*, 2025). The mechanism by which plants sense oxygen levels involve the use of oxygen as a cofactor by a class of enzymes known as plant cysteine oxidases (PCOs), which oxidize the N-terminal cysteine residue of specific target proteins, leading to their proteasomal degradation (Weits *et al.*, 2014; White *et al.*, 2017). This process affects the stability of several proteins, with the regulation of a subset of transcription factors, specifically members of the group VII ethylene response factors (ERF-VIIs), being critical for activating the expression of hypoxia-responsive genes (HRGs) that facilitate plant adaptation to low oxygen conditions (Gibbs *et al.*, 2011; Licausi *et al.*, 2011).

Although the mechanism by which oxygen modulates the transcription of HRGs through the stabilization and degradation of ERF-VIIs is well established, recent research has identified several additional factors that play significant roles in fine-tuning hypoxia responses. The rapid and efficient induction of HRGs at the onset of hypoxia is essential, but equally important is the subsequent modulation of this response to match the metabolic state of the plant, which is crucial for effective adaptation to hypoxic conditions. Upon reoxygenation, PCOs are expected to quickly trigger the degradation of ERF-VII proteins. However, although the transcriptional output of HRGs rapidly declines upon reoxygenation (Kosmacz *et al*., 2015), the underlying mechanism remains incompletely resolved.

In this review, we summarize the latest advances in our understanding of oxygen sensing, its activation, inactivation, and regulation, with a particular focus on the spatiotemporal dynamics of oxygen availability in plants.

## The oxygen-sensitive branch of the N-degron pathway

The ability of plants to detect and adapt to changes in oxygen supply can be primarily attributed to the N-degron pathway, in which transcriptional regulators are directly connected to cellular oxygen levels (Gibbs *et al.*, 2011; Licausi *et al.*, 2011). In *Arabidopsis thaliana*, ERF-VII transcription factors represent the best-characterized substrates of this pathway and control the transcriptional switch that enables plant survival under hypoxic stress (Loreti and Perata, 2023).

In contrast, additional substrates of the oxygen-sensitive N-degron pathway primarily function in developmental regulation. Among these, the Polycomb Repressive Complex 2 subunit VERNALIZATION 2 (VRN2) acts as a transcriptional repressor linking oxygen availability to developmental timing and meristem activity, including the regulation of flowering and root growth (Gibbs *et al.*, 2018). Similarly, the transcription factor LITTLE ZIPPER 2 (ZPR2) regulates shoot apical meristem activity and leaf initiation in response to endogenous hypoxia, thereby coupling oxygen sensing to morphogenetic processes (Weits *et al.*, 2019).

While these substrates highlight the developmental versatility of the pathway, the canonical response to acute oxygen deprivation relies on ERF-VIIs. Through the regulation of a defined set of HRGs, ERF-VIIs reprogramme plant metabolism and growth to sustain cellular homeostasis under oxygen deprivation (Gibbs *et al.*, 2011; Licausi *et al.*, 2011).

In air, ERF-VII proteins are subject to turnover via the oxygen-dependent branch of the N-degron pathway (Fig. 1). The initiator methionine is removed, exposing the N-terminal cysteine, which is oxidized to cysteinesulfinic acid by PCOs, with molecular oxygen serving as a co-substrate (Weits *et al.*, 2014). The cysteinesulfinic acid is then used as a substrate for arginyl-tRNA-protein transferases (ATEs), which add an arginine to the N-terminus. This arginylated protein is then recognized by the E3 ubiquitin ligase protein PRT6 and subsequently degraded through the proteasome (Gibbs *et al.*, 2011; Licausi *et al.*, 2011). As a result, when oxygen concentration is low, the activity of PCOs is reduced, and oxidation and subsequent degradation are prevented; ERF-VII proteins are stabilized and ultimately accumulate within the nucleus and trigger the induction of HRGs (Fig. 1) (Weits *et al.*, 2014).

**Fig. 1.**
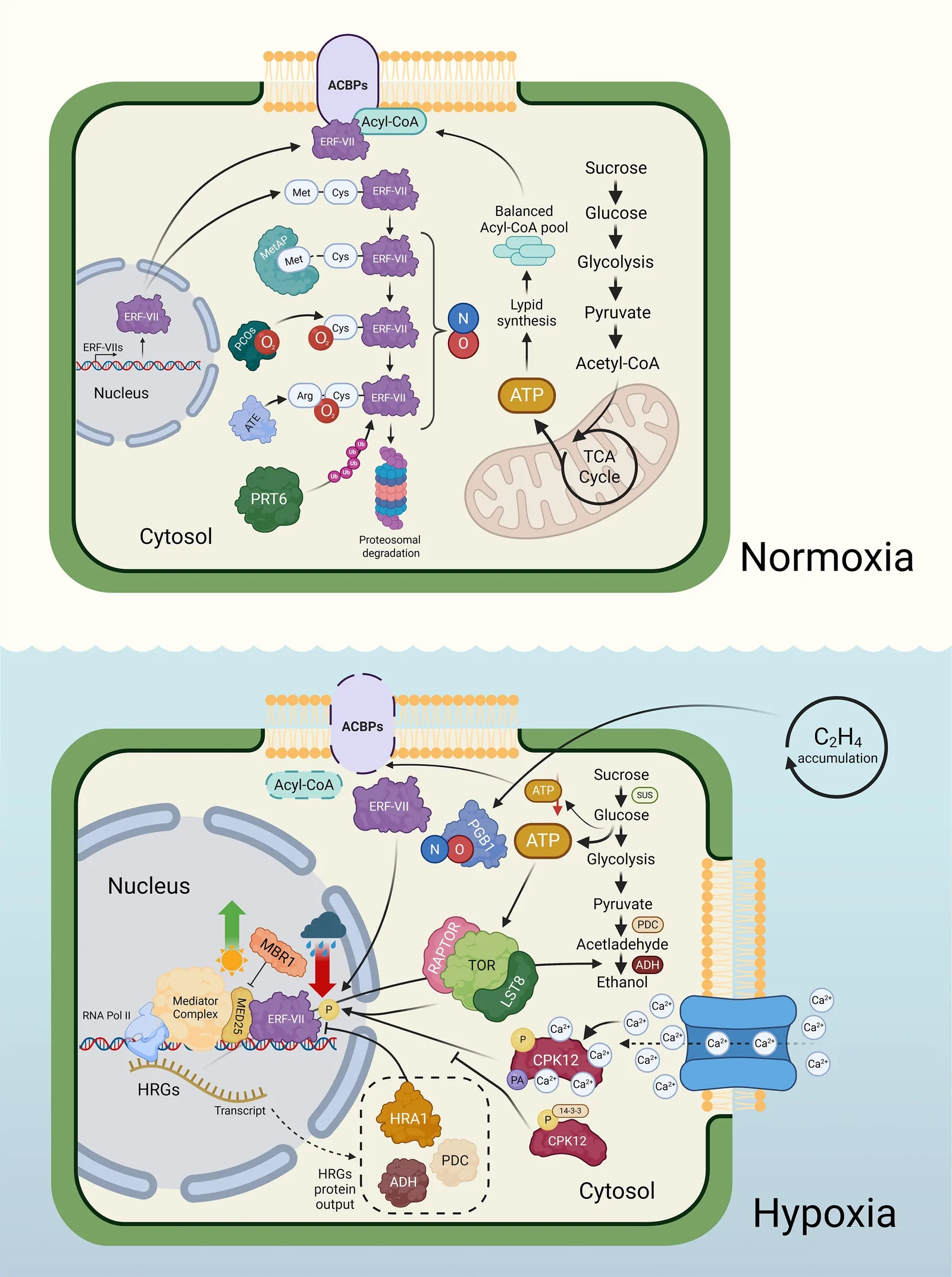
Molecular pathway activating the response to hypoxia through oxygen sensing. Top: under normoxic conditions, ERF-VII transcription factors are continuously synthesized and undergo rapid turnover via the N-degron pathway. A fraction of newly synthesized ERF-VII proteins can associate with acyl-CoA-binding proteins (ACBPs), generating a dynamic membrane-associated pool that co-exists with ongoing proteasomal degradation. In the cytosol, the N-terminal Met–Cys of ERF-VIIs undergoes processing followed by oxidation by plant cysteine oxidases (PCOs), and then by arginylation and subsequent proteasomal degradation via the PRT6 pathway. Nitric oxide (NO) is shown as a modulatory redox signal influencing ERF-VII stability. The bracket indicates that NO impacts the N-degron pathway without implying a defined or exclusive molecular target, as the precise level at which NO acts within the pathway remains unresolved. Under normoxia, sugar-derived carbon enters glycolysis and the tricarboxylic acid (TCA) cycle via mitochondrial respiration, ensuring ATP production through oxidative phosphorylation. Bottom: as oxygen levels decrease, multiple mechanisms converge to activate the anaerobic response. ERF-VIIs are released from the plasma membrane, facilitating their nuclear translocation. Once in the nucleus, ERF-VIIs interact with the MED25 subunit of the multiprotein Mediator complex to drive the optimal transcription of hypoxia-responsive genes (HRGs). Plants growing in environments with excessive rainfall, natural variation in MBR1 sequence is observed and its activity is reduced, thereby enhancing the function of MED25. In parallel, the TOR kinase acts as a metabolic checkpoint by phosphorylating conserved serine residues on RAP2.12, thereby fully activating HRGs when ATP levels and carbon availability are sufficient. Simultaneously, hypoxia triggers rapid accumulation of cytosolic Ca^2+^, which precedes transcriptional changes, with CPK12-dependent phosphorylation further stabilizing ERF-VIIs. Additionally, the translocation of CPK12 from the cytosol to the nucleus is modulated by lipid signalling and protein interactions: phosphatidic acid acts as a positive regulator of its nuclear import, while the 14-3-3κ protein restricts this translocation. This dynamic regulation establishes a CPK12–ERF-VII module that converts Ca^2+^ signals into the transcriptional activation of HRGs, effectively linking cytosolic sensing to nuclear hypoxia signalling. Simultaneously. phosphorylated CPK12 engages a negative feedback mechanism, probably involving 14-3-3-mediated regulation, to limit its own activity and ensure tight temporal control of Ca^2+^-dependent hypoxia signalling. When hypoxia originates from submergence, ethylene is produced and accumulates, inducing phytoglobin (PGB) expression. PGB promotes NO scavenging, accelerating ERF-VII stabilization. Fine-tuning of the pathway is achieved by HRA1, which interacts with RAP2.12, reducing its ability to bind to HRG promoters. This feedback inhibition limits excessive ERF-VII activity, conserving energy and facilitating the transition towards post-hypoxic recovery. Created in BioRender. Castellana, C. (2026) https://BioRender.com/890rlue.

Comparative genomics has recently shown that ERF-VIIs are a tracheophyte innovation that repurposed an ancient redox-sensitive proteolytic system into a dedicated oxygen-sensing switch (Dalle Carbonare *et al.*, 2025). While the enzymatic core of the N-degron pathway operating in oxygen sensing (PCO–ATE–PRT6) is broadly conserved within land plants, canonical ERF-VII transcription factors carrying the N-terminal Met–Cys motif are restricted to vascular plants and are absent from algae and bryophytes. In their place, earlier land plants possess unrelated Cys_2_-ERFs that can be targeted for N-degron-mediated degradation but not stabilized by hypoxia (Dalle Carbonare *et al.*, 2025). Canonical ERF-VIIs first appeared at the evolutionary emergence of roots and vascular tissues—oxygen-limited habitats—and diversified phylogenetically by optimizing the N-terminal MCGGAI/V motif to enhance the binary oxygen-sensing behaviour of angiosperm ERF-VIIs. Functional studies confirm that only canonical ERF-VIIs mediate the activation of hypoxic-responsive promoter sequences with HRPE and GCC-box motifs, respectively. Overall, this suggests a co-evolution of ERF-VII sequence, promoter architecture, and physiological need. Importantly, these studies position ERF-VIIs as a primary evolutionary innovation allowing vascular plants to sense oxygen availability to modify transcription (Dalle Carbonare *et al.,* 2025).

The PCO/PRT6 module acting on ERF-VII transcription factors and VRN2/ZPR2 thus constitutes the canonical plant oxygen-sensing pathway. It integrates both oxygen and redox signals leading to transcriptional reprogramming. This module represents the first tier of the entire hypoxia signal network, which is subsequently refined by nitric oxide (NO), membrane anchoring, metabolic cues, calcium signalling, and transcriptional co-regulation mechanisms, as shown in the subsequent sections.

## Membrane anchoring and acyl-CoA-binding protein-mediated release

While the N-degron pathway determines the stability of ERF-VII transcription factors in an oxygen-dependent manner, there is an additional level of regulation that controls their cellular localization and ultimately their access to the nucleus. Under normoxic conditions it has been shown that RAP2.12 is localized at the plasma membrane due to direct interactions with acyl-CoA-binding proteins (ACBPs), namely ACBP1 and ACBP2 (Licausi *et al.*, 2011). Both peripheral membrane proteins contain ankyrin-repeat domains that associate the ERF-VIIs with the cytoplasmic aspect of the plasma membrane, thus sequestrating them from the nuclear transcriptional machinery (Fig. 1).

This process of membrane anchoring acts as a ‘safety lock’ for the hypoxia transcriptional programme by preventing premature activation under normoxia, and possibly also representing a store of ERF-VII proteins which is ready to be released and translocated to the nucleus to activate the promoters of HRGs. When the cell is deprived of oxygen, the ERF-VII proteins are released from the ACBP complex and translocate to the nucleus (Licausi *et al.*, 2011). The exact molecular trigger for this release is still debated. However, earlier investigations using biochemical assays and confocal imaging determined that the release event coincides with a decreased cellular energy charge and a corresponding depletion in ATP content, which indicates that energetic stress may provoke dissociation of the ERF–ACBP interaction (Fig. 1) (Schmidt *et al.*, 2018).

Importantly, this mechanism does not substitute the rapid oxygen-dependent stabilization of the ERF-VIIs mediated by the N-degron pathway, which occurs early upon hypoxia and precedes the induction of the HRGs (Zubrycka *et al.*, 2023). Rather, ERF-VII membrane association via ACBPs may represent a complementary regulatory layer, potentially modulating the subcellular availability, spatial distribution, or effective concentration of ERF-VIIs. In this context, ACBP-mediated sequestration could function as a fine-tuning mechanism, contributing to the robustness and control of hypoxia-responsive transcription rather than acting as a prerequisite for its initiation.

Supporting this model, ACBPs are recognized to interact with long-chain acyl-CoA esters, key intermediates in lipid metabolism whose accumulation is determined by energy status. Reductions in mitochondrial activity and changes in the flux of fatty acids, as a result of hypoxia, can change binding between ACBP and the ligand, thus bringing about conformational changes that decrease the affinity of ACBP for RAP2.12 and move it to the nucleus (Schmidt *et al.*, 2018). The dynamic interaction of ACBP and ERF-VIIs is reversible: reoxygenation re-establishes the membrane localization of RAP2.12, so that the oxygen-sensing system can be rapidly reset once normoxia is achieved (Licausi *et al.*, 2011).

Fluorescent protein imaging of Arabidopsis transgenic lines or protoplasts has captured the dynamics of this process, as shown by the relocalization of green fluorescent protein (GFP)-tagged RAP2.12, from the plasma membrane to the nucleus within minutes of the onset of hypoxia (Licausi *et al.*, 2011). This spatio-temporal plasticity allows the ERF-VII system to assess both oxygen availability and the energy status of the cell, such that transcriptional activation preferentially occurs when oxygen limitation coincides with an unmet demand for mitochondrial ATP production. Oxygen and energy availability appear to work together to regulate the threshold of ERF-VII activation. Under normoxia, high ATP levels support active lipid metabolism and keep long-chain acyl-CoA esters bound to ACBPs, which stabilizes the conformation of the ACBPs and drives the affinity for RAP2.12 or other ERF-VIIs (Schmidt *et al.*, 2018). When oxygen is limited, the rate of mitochondrial respiration slows down, ATP levels decrease, and lipid homeostasis is disrupted. Each disruption in metabolism will probably change the affinity for binding acyl-CoA and may result in small conformational changes in the model ACBPs, weakening associations with ERF-VIIs and promoting nuclear translocation (Schmidt *et al.*, 2018).

The control of the localization of ERF-VIIs dependent on energy adds an extra layer of sensing, connecting oxygen perception to cellular metabolism, offering a reasonable mechanism for the nuclear re-localization of RAP2.12 (Licausi *et al.*, 2011). Furthermore, pharmacological inhibition of ATP synthesis, or mutations caused by affected mitochondrial activity, represents energy loss and stimulates ERF-VIIs stabilization and upstream hypoxia-induced response genes, supporting the mechanism being ATP dependent (Schmidt *et al.*, 2018). Consistently, mitochondrial retrograde signalling mediated by the uncoupling protein UCP1 has been shown to inhibit the PCO branch of the N-degron pathway independently of external hypoxia, thereby stabilizing ERF-VII substrates and activating hypoxia-associated transcriptional programmes (Barreto *et al.*, 2022). The low ATP-dependent release of ERF-VIIs links energy status as a metabolic rheostat, that integrates oxygen cues, redox shifts, and lipid monitoring; all these varied effector signal outputs become a coherent signal. By integrating low-oxygen perception with declining cellular energy, plants effectively ration hypoxia responses to situations where low oxygen and metabolic stress are coupled, a central tenet of the multilayered ‘switching ON’ process (Fig. 1).

Importantly, stabilization of ERF-VII proteins in response to hypoxia is extremely rapid and precedes the induction of the HRGs (Zubrycka *et al.*, 2023). The induction of HRGs is thought to be triggered by the combined action of rapidly stabilized pre-existing ERF-VIIs, including RAP2.12, and *de novo* synthesis of RAP2.12 and other ERF-VII proteins (Licausi *et al.*, 2011; Kosmacz *et al.*, 2015), while ACBP-mediated membrane association is proposed to provide an additional energy-dependent regulatory layer rather than acting as a primary trigger of transcriptional activation. While HRG induction is fast, and occurs within 1 h of hypoxia, the nuclear localization of RAP2.12 is apparently slower, being detectable only after 3 h (Kosmacz *et al.*, 2015). This delay could be attributed to a more rapid nuclear re-localization of ERF-VIIs other than RAP2.12, such as RAP2.2/RAP2.3, or to the fact that only a small quantity of RAP2.12, undetectable by the methods used, is sufficient for activating HRG transcription (Kosmacz *et al.*, 2015). Although the relative contributions of *de novo* synthesized ERF-VIIs and ERF-VIIs released from ACBP remain unresolved, a decrease in ATP levels in plants exposed to hypoxia, as discussed above, serves as a trigger for RAP2.12 release from ACBP (Schmidt *et al.*, 2018). However, the release of ERF-VIIs from ACBP probably requires up to 4 h of hypoxia, since this is the time required to observe a 50% reduction in ATP content in the cell, a decrease that is required to significantly inhibit LONG-CHAIN ACYL-COA SYNTHETASE (LACS) activity (Schmidt *et al.*, 2018). Reduction by 50% of the ATP level by chemical inhibition of mitochondrial activity could only moderately induce some HRGs; therefore, together with the oleoyl-CoA-dependent release of RAP2.12 from ACBP, *de novo* ERF-VIIs synthesis is very likely to be required for a full anaerobic response at the transcriptional level.

## Target of rapamycin and the metabolic integration of oxygen sensing

The kinase target of rapamycin (TOR) is a master integrator of nutrient, energy, and oxygen status to coordinate plant growth and metabolic potential. In plants, TOR promotes biosynthetic and translational activity when conditions are energetically favourable, and inhibition of TOR activity occurs rapidly in response to lower ATP levels to devote metabolism to energy conservation and autophagy (Dobrenel *et al.*, 2016; Fu *et al.*, 2020).

Kunkowska *et al.* (2023) showed evidence that TOR couples energy availability to oxygen sensing to modulate the transcriptional activity of ERF-VII transcription factors in Arabidopsis (Fig. 1). Under adequate energy conditions, TOR phosphorylates two conserved serine residues (Ser346 and Ser352) on the C-terminus of RAP2.12 to fully activate HRGs. In contrast, under carbon or ATP starvation, TOR activity decreases, preventing RAP2.12 phosphorylation, and dampening the hypoxic transcriptional output. TOR inhibition, either genetically or pharmacologically, mimics carbon starvation and dampens HRG induction despite the presence of hypoxia-stabilized ERF-VIIs (Kunkowska *et al.*, 2023).

Thus, TOR connects perception of oxygen and energy signalling in cells. By phosphorylating RAP2.12, TOR also guarantees their transcriptional activity, and subsequent responses will occur when energy stores are sufficient to ensure that the costs of HRG induction are fully justified. In this context, it is important to highlight that several HRGs code for enzymes involved in carbohydrate metabolism and in the fermentative glycolytic steps. Low carbohydrate availability means that the substrates for glycolysis and fermentation are insufficient, and induction of enzymes involved in these pathways would represent a cost for the hypoxic cell that might compromise its viability. Indeed, it was demonstrated that the hypoxic response is significantly reduced when either sucrose or starch availability is limited (Loreti *et al.*, 2005, 2018). Notably, decreased TOR activity coincided with a decrease in ATP under submergence and a clear reduction in the phosphorylation of S6K, a ribosomal kinase that is one of the best-characterized direct substrates of TOR and is widely used as a molecular proxy for TOR activity. The loss of S6K phosphorylation therefore supports the role of TOR as a quick-acting energy rheostat during oxygen deprivation (Fig. 1). (Kunkowska *et al.*, 2023).

TOR signalling brings together oxygen, sugar, and ATP sensing into a single regulatory system. By integrating all of this via its modulation of activation of ERF-VIIs and global metabolism, TOR ensures that the rapidity of low-oxygen responses is not just oxygen dependent, but metabolically sustainable.

## Phytoglobins and nitric oxide: modulating oxygen and redox balance

In addition to regulation of the N-degron pathway branch which is dependent on oxygen, plants use phytoglobins (PGBs) and NO for additional regulation of the hypoxic response through a redox module, with a direct link to ERF-VII stability (Hebelstrup *et al.*, 2012; Gibbs *et al.*, 2014; Hartman *et al.*, 2019). PGBs, originally termed non-symbiotic haemoglobins, function as high-affinity oxygen-binding proteins which can scavenge NO via the PGB–NO cycle (Igamberdiev, 2004; Dordas and Hill, 2006). ). In this cycle, PGBs act as NO scavengers by catalysing its oxidation to nitrate. This limits NO accumulation under hypoxia and, in turn, restrains NO-dependent activation of the N-degron pathway, contributing to ERF-VII stabilization (Gibbs *et al.*, 2014; Hartman *et al.*, 2019). Crucially, it has been demonstrated that passive ethylene entrapment during early submergence up-regulates *PGB1*, driving this NO depletion even before oxygen levels fall critically low. This ethylene-mediated signalling enhances ERF-VII stability, effectively priming the plant to survive hypoxic stress (Hartman *et al.*, 2019). Through the dynamic interaction of oxygen, NO, and PGBs, the activation of hypoxia signalling is intimately tied to the redox state of the cell, which regulates ERF-VII stability and primes the transcriptional response to stress (Gibbs *et al.*, 2014; Hartman *et al.*, 2019).

Therefore, PCOs are not the only determinant of rapid ERF-VII-dependent activation of HRG expression. Rather, additional mechanisms contribute to ensure a rapid transcriptional response, safeguarding the plant from being killed by the sudden lack of oxygen availability. NO levels need to be concomitantly reduced, and the activity of PGB serves as a mechanism to accelerate the stabilization of ERF-VIIs. Remarkably, the fact that PGB is induced by ethylene links the PGB-dependent boosting of the ERF-VII pathway to hypoxia resulting from environmental factors, such as waterlogging and submergence, conditions in which ethylene entrapment in the submerged plant tissue leads to activation of ethylene sensing which includes PGB expression (Perata, 2020).

The activity of PCOs is repressed at oxygen concentrations as low as <3% (Puerta *et al.*, 2019), and the induction of HRGs is fast, within 30 min (Loreti *et al.*, 2005). Hypoxia generated by submergence after a flooding event does not arise instantly. Internal oxygen levels decrease progressively, and the activation of HRGs therefore requires more time to be established, presumably after a few hours (Perata, 2020). When plants are partially submerged, and oxygen levels are not yet low enough to ensure activation of HRGs, ethylene accumulates (Hartman *et al.*, 2019). This primes the submerged plant for the forthcoming hypoxic conditions, as ethylene signalling increasing the stability of ERF-VII proteins via PGB-directed scavenging of NO (Hartman *et al.*, 2019). The ethylene-dependent priming effect ensures faster activation of the ERF-VII pathway once hypoxia is established in submerged plants, contributing to their tolerance. Remarkably, the convergence of ethylene signalling and oxygen sensing allows the plant to discriminate between hypoxia caused by flooding, during which both ethylene entrapment and low oxygen occur, and chronic hypoxia that arises endogenously in specific plant tissues (Loreti and Perata, 2020).

## MBR1, MED25, and climate-dependent transcriptional reprogramming

A unique regulatory layer within the hypoxia signalling pathway has recently been discovered based on the identification of MBR1 (MED25-BINDING RING-H2 PROTEIN 1), which is an E3 ubiquitin ligase capable of regulating the stability of the Mediator complex subunit 25 (MED25) to modulate gene expression in an ERF-VII-dependent manner (Iñigo *et al.*, 2012; Castellana *et al.*, 2024). MED25 acts as a co-activator of transcriptional elongation to link transcription factors such as RAP2.12 and RAP2.2 with RNA polymerase II to produce the full hypoxic transcriptome (Ou *et al.*, 2011; Shukla *et al.*, 2019; Schippers *et al.*, 2024). MBR1 acts upstream of MED25, targeting it for proteasomal degradation, thereby regulating the magnitude of transcriptional activation at low oxygen levels (Fig. 1).

Castellana *et al.* (2024) utilized an environmental genome-wide association study (eGWAS) across 934 accessions of Arabidopsis to show that natural allelic variation in *MBR1* is associated with precipitation regimes, building a connection between *MBR1* and adaptation to rates of waterlogging and flooding. Accessions from wet habitats contained the *MBR1w*et allele, which has two missense variants that lower E3 ligase activity and subsequently improve the stability of MED25. Functional validation studies showed that *mbr1* knockout mutants have higher genetic induction of core hypoxia genes (*ADH1*, *PDC1*, *PCO1*, and *HRA1*) and higher tolerance to both waterlogging and submergence, while *med25* mutants demonstrated the opposite phenotype, emphasizing the role of *MBR1* as a repressor and MED25 as a much-needed component of hypoxia gene expression (Fig. 1).

Luciferase assays confirmed that MED25 is important for RAP2.12 and RAP2.2-mediated activation of anaerobic promoters, establishing that *MBR1* indirectly modulates the response to the oxygen-sensing cascade by regulating MED25 turnover. *MBR1* is transcriptionally down-regulated during hypoxia, releasing MED25 from the pressure of degradation, and permitting it to cooperate with ERF-VIIs in inducing transcriptional reprogramming to support life in an environment lacking oxygen.

MBR1, together with MED25, controls the magnitude of the hypoxic response rather than the speed at which HRGs are induced. MBR1 acts as a negative regulator of the hypoxic response upstream of MED25, and highlights how natural allelic variation, such as that exemplified by *MBR1wet*, enables adaptive fine-tuning of ERF-VII-mediated transcriptional responses in populations exposed to recurrent flooding in climates characterized by heavy and frequent precipitation events.

## Calcium as a fast secondary messenger

Several studies have suggested that Ca^2+^ signalling is one of the fastest and most integrative elements in the plant response to hypoxia (Bakshi and Gilroy, 2025). In Arabidopsis, for example, a decrease in oxygen concentration to <5% resulted in rapid spikes in cytosolic Ca^2+^, and these spiking events occurred prior to transcriptional reprogramming and metabolic changes, all in seconds. Ca^2+^ signals arise from both cellular influx across the plasma membrane, via cyclic nucleotide-gated channels (CNGCs) and reactive oxygen species (ROS)-activated channels such as HPCA1, and intracellular stores, particularly vacuoles, which can signal through cation/H^+^ exchanger (CAX) antiporters and autoinhibited calcium ATPases (ACAs) (Bakshi *et al.*, 2023; Fan *et al.*, 2023). One important vacuolar transporter gene, *CAX2* (*CATION EXCHANGER 2*), is a critical regulator of the response: *CAX2* loss-of-function mutants displayed elevated cytosolic Ca^2+^ levels even under normoxic conditions, which preceded the activation of HRGs and were also correlated with flooding tolerance (Bakshi *et al.*, 2023). Further downstream, Ca^2+^-dependent protein kinases (CPKs) and calmodulin-like proteins (CMLs) have been implicated to translate these ionic shifts to the transcriptional outputs. CPK12, for example, directly phosphorylates and stabilizes the hypoxia-activated ERF-VII transcription factors RAP2.3 and RAP2.12, allowing for their nuclear accumulation to trigger HRG activation (Fig. 1) (Fan *et al.*, 2023). Recent findings also suggests that CPK12 does not maintain a consistent presence in the nucleus and instead its presence is regulated by phosphatidic acid and 14-3-3κ proteins. Phosphatidic acid promotes the translocation of CPK12 into the nucleus, while 14-3-3κ prevents this event by restraining CPK12 in the cytoplasm. This dual regulation establishes a finely tuned CPK12–ERF-VII module that converts Ca^2+^ fluctuations into a transcriptional output, providing a molecular conduit through which cytoplasmic signals are transduced into a hypoxia-responsive nuclear programme (Fig. 1) (Fan *et al.*, 2023). *CML38*, again, reacts to oxygen stress by localizing stress granules to facilitate selective mRNA turnover and reallocation of resources via cytosolic and vacuolar autophagic pathways (Field *et al.*, 2021). These examples illustrate how Ca^2+^ signalling connects the sensing of membrane perturbation to transcriptional regulation.

More recently, the vacuolar H^+^/Ca^2+^ exchanger gene *CAX1* (*CATION EXCHANGER 1*) has been identified as a key regulator of the calcium signaling pathway (Yang *et al.*, 2022). *CAX1* loss protects against anoxia and submergence, as Ca^2+^ fluxes are regulated during the hypoxia and reoxygenation phases, reducing ROS spikes and changes in HRG expression (Yang *et al.*, 2022). The *cax1* mutants even show partial activation of ERF-VII target genes in normoxic conditions, suggesting that Ca^2+^ homeostasis occurring at the tonoplast primes the transcriptional machinery in response to low oxygen stress (Yang *et al.*, 2022). This implies that vacuoles not only serve as Ca^2+^ reservoirs, but are also involved both in oxygen sensing and in the recovery from hypoxic stress.

## Switching off the hypoxic response: reoxygenation as a multilayered event

In natural environments, hypoxia is usually transient; after submergence, as the water level recedes, plant tissues are progressively reoxygenated. The reoxygenation phase after de-submergence is critical for plant survival. Indeed, plants need to cope with a combination of oxidative, photo-oxidative, and dehydration stresses arising from the rapid re-exposure to oxygen and light (Yeung *et al.*, 2019; Pucciariello and Perata, 2021). Upon reoxygenation, plants are required to switch back from a fermentative metabolism to aerobic respiration to sustain plant energy demand. A sustained anaerobic respiration under normoxic conditions would result in the inefficient use of carbohydrates and is to be avoided in order to ensure rapid recovery from hypoxia (Paul *et al.*, 2016). Furthermore, the expression of genes required under hypoxia is presumably detrimental for normal, aerobic plant growth. To ensure an efficient transition to air, plants must rapidly repress the transcription of HRGs. Hypoxic transcripts return to basal levels within 2 h of reoxygenation (Kosmacz *et al.*, 2015; Weits *et al.*, 2023). These findings indicate that plants rapidly perceive a sudden rise in oxygen levels and consequently repress the transcription of HRGs. Given the rapid decay of hypoxia-responsive transcripts, an equally fast degradation of the transcription factors driving their expression would be expected. However, several studies showed that the stability of the ERF-VII transcription factors upon reoxygenation is not as fast as that of their target genes. The signal of RAP2.12 fused to GFP completely disappears from the nucleus only 3–4 h after reoxygenation (Kosmacz *et al.*, 2015). More recently, a luminescence signal from the first 28 amino acids of RAP2.12 fused to firefly luciferase (*Fluc*) was observed up to 3 h after reoxygenation (Brunello *et al.*, 2025). RAP2.12 and RAP2.3 were also found to be stable and localized in the nuclei up to 3 h after reoxygenation (Akter *et al.*, 2024, Preprint). However, a markedly faster decline of ERF-VIIs upon reoxygenation has been reported under specific experimental conditions. In particular, RAP2.3 levels were shown to return to baseline within 30–60 min after reoxygenation in plants grown under continuous light and exposed to a short hypoxic treatment (60 min) (Zubrycka *et al.*, 2023). These observations suggest that the kinetics of ERF-VII degradation upon reoxygenation might be influenced by the duration and intensity of the hypoxic stress, as well as by growth conditions, probably reflecting differences in the size of the stabilized protein pool accumulated during hypoxia.

The temporal discrepancy between HRG expression and ERF-VII protein stability suggests a regulatory mechanism during the reoxygenation phase that is more complex than the expected, PCO-dependent degradation of ERF-VIIs. Remarkably, two of the five PCO enzymes, namely PCO1 and PCO2, are transcriptionally activated by hypoxia itself, and are localized in nuclei (Weits *et al.*, 2014). Given that without oxygen PCOs would be unable to be enzymatically active, it is tempting to speculate that hypoxia-inducible PCOs represent a mechanism for ensuring a rapid oxidation of the N-terminal Cys residue of ERF-VIIs in the nuclei themselves. The above-reported evidence of relatively stable ERF-VII proteins upon reoxygenation indicates that this assumption is presumably wrong. ERF-VII stability upon reoxygenation is possibly sustained by ROS, which accumulate during the recovery phase (Fig. 2) (Akter *et al.*, 2024, Preprint). Upon reoxygenation, the restoration of normoxic conditions triggers a rapid burst of ROS generation through multiple pathways. These include the sudden reactivation of mitochondria, whose electron transport chain components are partially impaired by hypoxia, as well as the activation of the plasma membrane NADPH oxidase RESPIRATORY BURST OXIDASE HOMOLOG D (RBOHD) (Chang *et al.*, 2012; Yeung *et al.*, 2018). Importantly, ROS act as inhibitors of PCOs, and their accumulation during reoxygenation delays the canonical N-degron-mediated degradation of ERF-VIIs, thereby prolonging their stability (Akter *et al.*, 2024, Preprint).

**Fig. 2.**
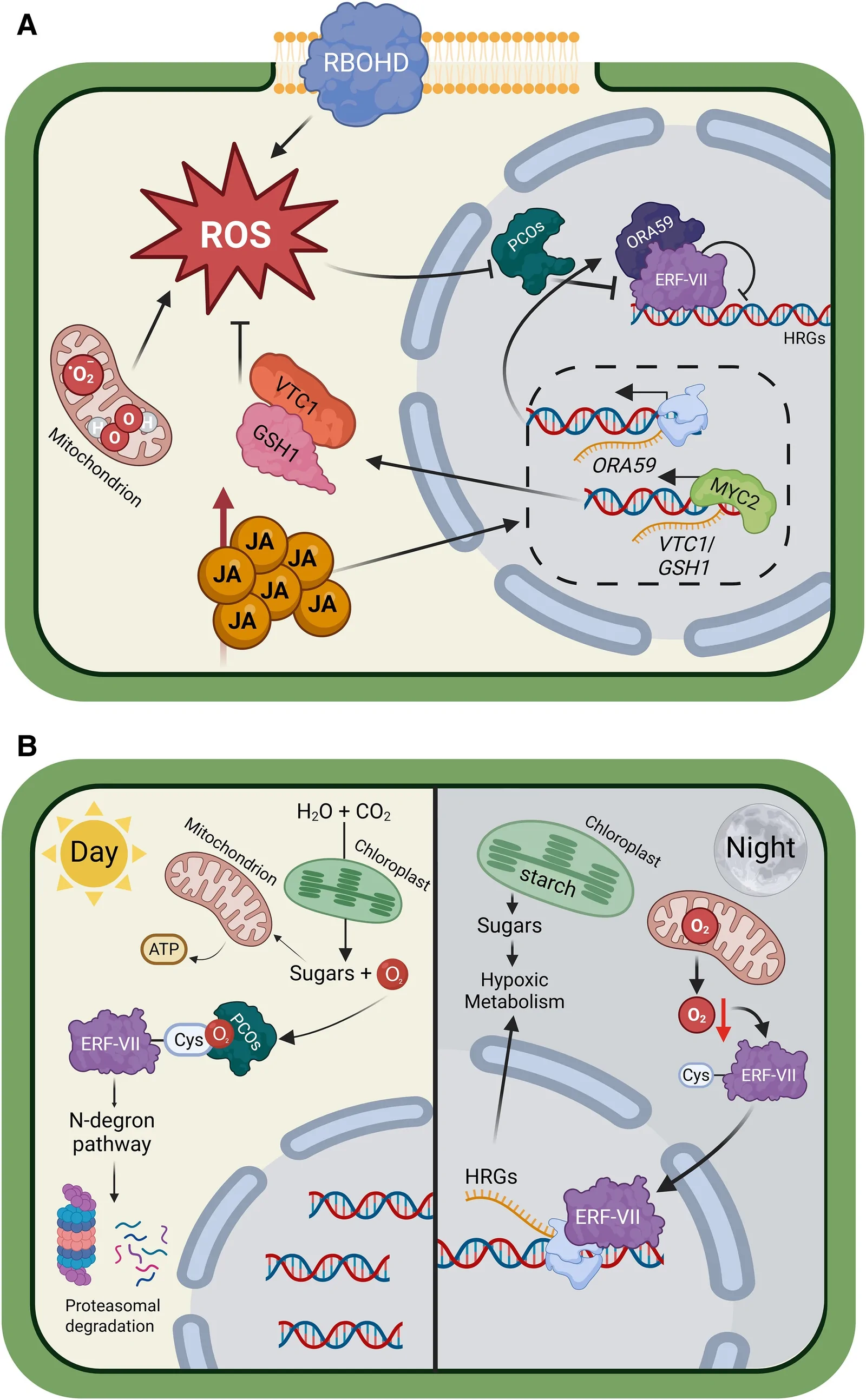
Reoxygenation signalling and day–night dynamics of oxygen sensing. (A) Reoxygenation after hypoxia triggers a rapid burst of reactive oxygen species (ROS) and a concomitant increase in jasmonate (JA) biosynthesis. ROS are generated both through RESPIRATORY BURST OXIDASE HOMOLOG D (RBOHD) activity at the plasma membrane and via the sudden reactivation of a hypoxia-damaged mitochondrial electron transport chain, which leads to electron leakage. ROS inhibit the activity of PCOs, preventing the canonical N-degron-mediated degradation of ERF-VII proteins. Despite remaining nuclear, ERF-VIIs appear to switch from transcriptional activators to repressors of HRGs. At the same time, JA accumulation activates the transcription factor MYC2, which binds to the promoters of antioxidant genes such as *VTC1* and *GSH1*, promoting their expression. JA also induces the expression of ORA59, which interacts with ERF-VII proteins and further limits their capacity to activate HRG transcription. (B) Throughout the day–night cycle, plants dynamically adjust their metabolic processes in response to fluctuations in oxygen availability. During the night, when light-dependent photosynthetic activity ceases, oxygen levels within plant tissues gradually decrease. This decline in oxygen concentration leads to the stabilization of ERF-VII proteins, as reduced oxygen levels inhibit the activity of PCOs, which normally degrade ERF-VIIs under normoxic conditions. As a result, stabilized ERF-VIIs accumulate in the nucleus, where they act as transcriptional activators, initiating the expression of HRGs that enable the plant to adapt to low-oxygen conditions. In contrast, during the day, the availability of oxygen increases as photosynthesis resumes, leading to the normalization of oxygen levels within the plant. This increase in oxygen concentration triggers a shift in energy production from anaerobic processes, such as fermentation, to the more efficient mitochondrial oxidative phosphorylation pathway, which generates ATP through aerobic respiration. As oxygen levels rise, the stability of ERF-VII proteins is compromised, and they undergo degradation through the canonical N-degron pathway. Created in BioRender. Castellana, C. (2026) https://BioRender.com/nc176tr.

However, this evidence does not solve the incongruence between the rapid decay of the transcripts of HRGs and the stability of ERF-VIIs during reoxygenation. Interestingly, in reoxygenated Arabidopsis plants after hypoxia, ERF-VIIs remain bound to the promoters of HRGs; however, despite their persistence, transcriptional repression of HRGs occurs (Akter *et al.*, 2024, Preprint). ERF-VIIs might therefore lose their ability to transactivate the promoters of HRGs and switch from activators to repressors of anaerobic gene expression (Akter *et al.*, 2024, Preprint). The burst of ROS generated upon reoxygenation, therefore, not only causes oxidative stress but also acts as an important signal cue (Fig. 2A) (Yeung *et al.*, 2018).

## Down-regulation of hypoxia signalling mediated by ERFVII repressors

Switching off the hypoxic response is not only mediated by the reoxygenation-dependent degradation of ERF-VIIs triggered by oxygen availability and PCO activity. Interaction of ERF-VIIs with proteins acting as repressors can lead to at least partial inactivation of the action of the ERF-VIIs on the promoters of HRGs. This scenario is compatible with the evidence of ERF-VIIs still present and bound to their target promoters during the reoxygenation phase (Akter *et al.*, 2024, Preprint). Inactivation or dampening of the ability of ERF-VIIs to activate transcription of HRGs was indeed reported. For instance, a gene belonging to the HRGs, namely *HYPOXIA RESPONSIVE ATTENUATOR1* (*HRA1*), is a repressor of the anaerobic response (Fig. 1) (Giuntoli *et al.*, 2014). HRA1 is induced by hypoxia and is localized in nuclei. Transcription of *HRA1* under hypoxia is accompanied by active loading of *HRA1* mRNA onto polysomes, indicating that the synthesis of HRA1 protein occurs during the stress (Giuntoli *et al.*, 2014). Experimental evidence demonstrated that HRA1 interacts with the RAP2.12 protein but binds only a few genomic DNA regions from hypoxia-regulated genes, indicating that HRA1 modulates RAP2.12 through protein–protein interaction (Giuntoli *et al.*, 2014). The trihelix protein encoded by *HRA1* interacts with RAP2.12, but not with the other four members of the ERF-VII family (Giuntoli *et al.*, 2014). Because of the interaction between HRA1 and RAP2.12, the activity of the latter is dampened, with a reduced expression of HRGs. The up-regulation of *HRA1* under hypoxic conditions thus serves as a feedback mechanism to limit RAP2.12 activity and prevent overactivation of low-oxygen-responsive genes, presumably to preserve energy from excessive consumption due to high transcription of HRGs (Fig. 1) (Giuntoli *et al.*, 2014, 2017). While the role of HRA1 to fine-tune the hypoxic response during oxygen deprivation has been demonstrated (Giuntoli *et al.*, 2014, 2017), there is no evidence for the role of HRA1 during reoxygenation.

Other factors may also be involved in the rapid repression of anaerobic gene expression once oxygen becomes available after hypoxia. Reoxygenation is accompanied by a burst in jasmonate (JA) synthesis, which plays multiple roles during recovery, including the activation of antioxidant defences that protect cells from ROS damage (Fig. 2A) (Yuan *et al.*, 2017). However, the mechanism underlying JA accumulation during reoxygenation remains unclear. Indeed, the accumulation of JA leads to the activation of the transcription factor MYC2 (Song *et al.*, 2022). As a consequence, MYC2 promotes the expression of *VITAMIN C DEFECTIVE* (*VTC*) and *GLUTATHIONE SYNTHETASE* (*GSH*), which encode enzymes involved in antioxidant defence (Yuan *et al.*, 2017). JA signalling also triggers the induction of several transcription factors, including OCTADECANOID-RESPONSIVE ARABIDOPSIS 59 (ORA59). ORA59 is a member of the ERF family that interacts with certain ERF-VII proteins, specifically RAP2.12, RAP2.2, and RAP2.3 (Brunello *et al.*, 2025). This interaction restricts the transcriptional activity of ERF-VIIs, thus contributing to the inhibition of anaerobic gene expression upon reoxygenation (Brunello *et al.*, 2025). In *ora59* mutant plants, the degradation of hypoxic transcripts is delayed, confirming the regulatory role of ORA59 during the early phase of reoxygenation (Brunello *et al.*, 2025).

Interestingly, JA signalling is also a key feature of the plant response to pathogens (Yang *et al.*, 2007). During plant–pathogen interactions, a hypoxic microenvironment is established at the infection site (Gravot *et al.*, 2016; Valeri *et al.*, 2021; Deng *et al.*, 2025). However, only a limited subset of HRGs is induced by hypoxia as a consequence of pathogen infection (Brunello *et al.*, 2025). ORA59 is induced during *Botrytis*-induced hypoxia, and its interaction with RAP proteins partially represses their activity. As a result, only a few HRGs are induced (Brunello *et al.*, 2025).

The interaction between selected members of the ERF-VII family and proteins acting as repressors, such as HRA1, which only binds to RAP2.12, and ORA59, which binds to RAP2.12, RAP2.2, and RAP2.3, suggests that these proteins act to fine-tune the hypoxic response. While experimental evidence suggests that HRA1, which is itself induced by the ERF-VIIs, mostly acts as a repressor in a feed-back mechanism for regulating the hypoxic response, ORA59 might also be involved in the switch-off of the ERF-VII mediated up-regulation of HRGs, although further evidence is required to confirm this hypothesis.

## Dynamic activation and inactivation of hypoxia signalling

Plants not only possess hypoxic niches in otherwise fully aerobic plant tissues, but also undergo endogenous, cyclic changes in oxygen availability, that are necessary for normal development (Triozzi *et al.*, 2024). Cyclic hypoxia is due to elevated mitochondrial respiration in actively expanding leaf primordia which is not compensated for by photosynthetic oxygen production during the night, causing a predictable and developmentally patterned fluctuation in internal oxygen levels (Fig. 2B) (Triozzi *et al.*, 2024). This balance drives a mechanism that regulates the utilization of sugar reserves during the night. The occurrence of cyclic hypoxia in young leaves is crucial for plant growth, underscoring the role of oxygen as a signalling molecule in plant development (Weits *et al.*, 2019). The underlying mechanism of cyclic hypoxia may involve the TOR-dependent pathway, which is responsible for energy sensing and signalling under low-oxygen conditions (Kunkowska *et al.*, 2023). Pharmacological inhibition of TOR activity attenuates cyclic hypoxia responses in young leaves during the night (Triozzi *et al.*, 2024). TOR inhibition could also limit energy consumption, thus limiting respiration rates and impairing the establishment of cyclic hypoxia, which is predominantly due to an excess of oxygen consumption during the night in the rapidly growing young leaves (Fig. 2B).

Cyclic HRG expression relies on ERF-VIIs, with continuous light and/or nocturnal hyperoxia disrupting the fluctuation in the expression of HRGs over the day/night cycle, highlighting its independence from the endogenous circadian clocks (Triozzi *et al.*, 2024). ERF-VII proteins become transiently stabilized during the night and activate the expression of HRGs. Cyclic hypoxia is severely dampened in a pentuple ERF-VII mutant, reinforcing the dependence of cyclic hypoxia on ERF-VII stabilization. Interestingly, strong stabilization of ERF-VIIs was observed during the night because of cyclic hypoxia (Triozzi *et al.*, 2024). This, even though the level of hypoxia during the night is mild (∼10%), is mirrored by an induction of HRGs which is evident but much lower than when the same tissues were exposed to 1% oxygen (Triozzi *et al.*, 2024), suggesting that besides ERF-VIIs other activators of the hypoxic response synergistically promote HRG expression under severe hypoxia.

## Concluding remarks

The mechanism by which plants sense hypoxia is largely understood to involve the oxygen-sensitive instability of ERF-VII transcription factors, as extensively characterized in *A. thaliana* under controlled experimental conditions (Fig. 1). While this mechanism is sufficient to elicit an appropriate response to hypoxic conditions, experimental data gathered over the past decade suggest that several additional components are required to fine-tune the molecular response of plants to limited oxygen availability. This fine-tuning occurs in terms of both the temporal dynamics of the response and its amplitude.

The activation of the signalling cascade leading to the expression of HRGs is rapid, with some HRGs being induced by hypoxia in <10 min (Loreti *et al.*, 2005; Puerta *et al.*, 2019). This rapidity probably reflects the convergence of multiple regulatory inputs on ERF-VII activity, including swift N-degron-dependent stabilization and nuclear accumulation, and modulatory layers that couple transcriptional output to metabolic state. While the dynamics of this process have been studied, there is still no conclusive explanation regarding the relative contributions of release of ERF-VIIs from ACBPs and *de novo* synthesis and stabilization of ERF-VIIs. Furthermore, the inability of PCOs to act under hypoxic conditions adds another layer of complexity. It is evident that variations in the enzymatic properties of PCOs and their expression patterns are likely to contribute to the adequacy of the response across different plant tissues and oxygen concentrations (Taylor-Kearney and Flashman, 2022).

The MBR1/MED25 complex has emerged as a key player in the oxygen-sensing mechanism, enhancing ERF-VII activity (Castellana *et al.*, 2024; Schippers *et al.*, 2024). Evidence suggests that plant adaptation to environments characterized by varying precipitation frequencies is facilitated by different MBR1 alleles, which confer increased tolerance to wet conditions. This represents one example of the finely tuned environmental adaptation mechanisms that have evolved to cope with naturally limited oxygen availability, ensuring that hypoxia responses are precisely calibrated to the ecological niche of each population (Holdsworth *et al.*, 2025).

Additionally, phosphorylation of ERF-VIIs appears to enhance the activity of these transcription factors, either through the calcium spike that occurs at the onset of hypoxia or via the action of the TOR pathway, which links energy sensing to oxygen availability (Fan *et al.*, 2023; Kunkowska *et al.*, 2023).

Upon reoxygenation following hypoxia (Fig. 2A), PCO activity is restored, potentially leading to the degradation of ERF-VIIs. This uncoupling suggests that ‘switching OFF’ is an active, multilayered process rather than a simple reversal of the ON mechanism. The delayed degradation of ERF-VIs upon reoxygenation may be due to the action of ROS, which are produced during reoxygenation and act as inhibitors of PCO activity, thus prolonging the presence of ERF-VIIs on HRG promoters (Akter *et al.*, 2024, Preprint). Although ERF-VIIs remains bound to HRG promoters during reoxygenation, a mechanism not yet fully understood prevents the activation of HRG transcription (Akter *et al.*, 2024, Preprint). In this context, repressive factors such as HRA1 and ORA59, which directly interact with and inhibit ERF-VII activity, may play a significant role (Giuntoli *et al.*, 2014; Brunello *et al.*, 2025).

Over a decade after the discovery of the oxygen-sensing mechanism in plants, our understanding of hypoxia signalling has significantly advanced. Nevertheless, much remains to be uncovered, as hypoxia in plants is a complex and multifaceted phenomenon. Its spatiotemporal occurrence not only influences adaptation to adverse environmental conditions but also extends to plant development and plant–microbe interactions, requiring sophisticated regulation to achieve the desired outcome.

Notably, the advent of high-resolution approaches such as single-cell and single-nucleus transcriptomics is beginning to uncover previously inaccessible layers of hypoxia biology. Single-nucleus analyses have revealed strong cell-type-specific and spatially resolved hypoxic responses within plant tissues, highlighting the heterogeneity of oxygen sensing and signalling at the cellular level (Hill *et al.*, 2026). Complementarily, single-cell approaches have provided increased resolution of hypoxia-associated developmental programmes, uncovering cell-type-specific layers of regulation underlying cell fate specification and tissue patterning (Castellana *et al.*, 2026).

Together, these advances position hypoxia signalling as a central integrative framework linking environmental sensing, metabolic state, and developmental decision-making, and highlight the need for future studies that bridge molecular mechanisms with cellular and organismal physiology in natural contexts.
